# Impact of mAb-FcRn affinity on IgG transcytosis across human well-differentiated airway epithelium

**DOI:** 10.3389/fimmu.2024.1371156

**Published:** 2024-09-16

**Authors:** Kohei Togami, Whitney Wolf, Lucas C. Olson, Madison Card, Limei Shen, Alison Schaefer, Kenichi Okuda, Larry Zeitlin, Michael Pauly, Kevin Whaley, Raymond J. Pickles, Samuel K. Lai

**Affiliations:** ^1^ Division of Pharmacoengineering and Molecular Pharmaceutics, Eshelman School of Pharmacy, University of North Carolina at Chapel Hill, Chapel Hill, NC, United States; ^2^ Marsico Lung Institute, University of North Carolina at Chapel Hill, Chapel Hill, NC, United States; ^3^ Department of Biomedical Engineering, University of North Carolina at Chapel Hill, Chapel Hill, NC, United States; ^4^ ZabBio, San Diego, CA, United States; ^5^ Department of Microbiology & Immunology, School of Medicine, University of North Carolina at Chapel Hill, Chapel Hill, NC, United States

**Keywords:** FcRn, monoclonal Abs, lung, airway, transcytosis, respiratory virus

## Abstract

Effective treatment and immunoprophylaxis of viral respiratory infections with neutralizing monoclonal antibodies (mAbs) require maintaining inhibitory concentrations of mAbs at the airway surface. While engineered mAbs with increased affinity to the neonatal Fc receptor (FcRn) are increasingly employed, little is known how increased affinity of Fc to FcRn influences basal-to-apical transepithelial transport (transcytosis) of mAbs across the airway epithelium. To investigate this, we utilized a model of well-differentiated human airway epithelium (WD-HAE) that exhibited robust FcRn expression, and measured the transepithelial transport of a mAb against SARS-CoV-2 Spike protein (CR3022) with either wildtype IgG_1_-Fc or Fc modified with YTE or LS mutations known to increase affinity for FcRn. Despite the marked differences in the affinity of these CR3022 variants for FcRn, we did not find substantial differences in basal-to-apical transport reflective of systemic dosing, or apical-to-basal transport reflective of inhaled dosing, compared to the transport of wildtype IgG_1_-Fc. These results suggest increasing FcRn affinity may only have limited influence over transcytosis rates of systemically dosed mAbs across the human airway epithelium over short time scales. Over longer time scales, the elevated circulating levels of mAbs with greater FcRn affinity, due to more effective FcRn-mediated recycling, may better resupply mAb into the respiratory tract, leading to more effective extended immunoprophylaxis.

## Introduction

Virus-laden aerosols and droplets can deposit in both the nasal passages of the upper respiratory tract (URT) as well as the major conducting airways of the lower respiratory tract (LRT), including the trachea and bronchi. Not surprisingly, both URT and LRT regions are common sites for the initiation of infections by many respiratory viruses, which is then followed by progressive spread of infection into the distal airways (bronchioles) and the alveolar lung regions (1). Specifically, continued spread of infection occurs via repeated cycles of apical virus infection and virus egress into the airway lumen, and is a hallmark feature of infection by many respiratory viruses, including respiratory syncytial virus (RSV) (2), influenza viruses (3), SARS-CoV-1 (4), SARS-CoV-2 (5), seasonal coronaviruses (6) and parainfluenza viruses (7). These respiratory viruses initiate infection exclusively via the apical surface of the airway columnar epithelial cells, with no productive infection if viruses are inoculated to the basolateral surface of an intact epithelium (4). More importantly, progeny virions traffic almost exclusively to the apical membranes of infected columnar epithelial cells from where they shed into airway mucus lining the airway lumen. Virions shed into airway mucus secretions face a variety of host factors, including a robust apical glycocalyx and variable amounts of immunoglobulins, that impact the continued spread of infection.

The luminal infection and spread of many respiratory viruses is consistent with the lack of viremia associated with most viruses early during infection, and why mucosal sampling via nasal swabs, nasal washes, or saliva remain the primary methods for identifying respiratory viral infections. It also underscores the need to deliver antivirals directly to the luminal surfaces of the respiratory tract (8, 9). Historically, therapeutics directed against respiratory viruses have relied on systemic administration including oral, intravenous, and intramuscular administration. This includes monoclonal antibodies (mAb), the first class of antiviral therapeutics to receive emergency use authorization (EUA) as early intervention against the progression of established SARS-CoV-2 infection to severe COVID-19 (10). To be efficacious, systemically dosed mAbs that reach the submucosal compartments must also undergo sufficient serosal to luminal transport to achieve effective neutralization levels at the airway surface and in airway secretions. Since the tight junctions formed between the luminal airway epithelial cells severely limits paracellular transport of many macromolecules from the serosal to luminal surface and vice versa (11–13), systemic mAbs must cross an intact respiratory epithelium by specific transcytotic mechanisms. The polymeric immunoglobulin receptor (PIGR) complex is one such mechanism for transcytosising mAbs, but is largely restricted to dimeric IgA interactions. Others have reported that the neonatal Fc receptor (FcRn) can mediate transcytosis of IgG through the vesicular transport pathway in the mucosal epithelium of the respiratory tract (14–19). While it has been generally assumed that increasing the affinity of the Fc region of IgG-based mAbs (IgG-Fc) to FcRn may further enhance the serosal to mucosal distribution of mAb, quantitative comparison of how FcRn affinity impacts serosal to mucosal transcytosis remains poorly defined.

To better understand whether IgG1-Fc affinity to FcRn affects the serosal to mucosal trancytosis of mAbs across human airway epithelium, we utilized well-differentiated cultures of human airway epithelium (WD-HAE) that reproduce the pseudostratified muco-ciliary columnar airway epithelium of the human respiratory epithelium *in vivo* (20–22). To elucidate how mAb Fc design impacted transcytosis in HAE, we engineered a panel of CR3022-based mAbs, which target the same receptor-binding domain (RBD) of both SARS-CoV-1 and SARS-CoV-2 (23), to have either: (i) the original IgG_1_-Fc (analogous to REGEN-COV and Regkirona); (ii) Fc containing the YTE mutation utilized in Evusheld (24) and Beyfortus (25); or, (iii) Fc containing the LS mutation utilized in Xevudy (26). In previous studies, both YTE and LS Fc mutations markedly extended the systemic circulation of mAbs due to more effective FcRn-mediated recycling in the liver (27, 28). Surprisingly, in contrast to the longstanding dogma that greater FcRn affinity would lead to increased transcytosis, we show here that greater FcRn-affinity does not appear to impact the rates of mAb transcytosis transport across the human respiratory epithelium.

## Materials and methods

### Production of mAbs in Nb7KOΔXylT/FucT N. benthamiana

Briefly, various CR3022 mAbs were expressed in *N. benthamiana* plants using “magnifection” procedure (29). Cloned expression vectors i.e., PVX-LC and TMV-IgG-HC were transformed into *Agrobacterium tumefaciens* strain ICF320 (Icon Genetics) and grown overnight at 28.0°CC followed by 1:000 dilution in infiltration buffer [10 mM MES (pH 5.5) and 10 mM MgSO_4_]. The combinations of diluted bacterial cultures (TMV-IgG-HC + PVX-LC) were used to transfect 4 week old *N. benthamiana* plants (ΔXTFT glycosylation mutants) using vacuum infiltration. Using a custom-built vacuum chamber (Kentucky Bioprocessing), the aerial parts of entire plants were dipped upside down into the bacterial/buffer solution and a vacuum of 24’’ mercury was applied for 2 min. Infiltrated plants were allowed to recover and left in the growth room for transient expression of antibodies. 7 days after infiltration, plants were harvested and homogenized in extraction buffer containing 100 mM Glycine, 40 mM Ascorbic Acid, 1 mM EDTA (pH 9.5) in a 0.5:1 buffer (L) to harvested plants (kg) ratio. The resulting green juice was clarified by filtration through four layers of cheesecloth followed by centrifugation at 10,000 g for 20 min. Next, mAbs were captured from the clarified green juice using MabSelect SuRe Protein A columns (GE Healthcare). The mAbs were eluted from Protein A columns were further purified using equilibrated Capto Q columns (GE Healthcare) and flow-through fractions, which contain mAbs, were collected. The mAb-containing fractions were finally polished with CHT chromatography with type II resin (Bio-Rad). The purity of the different mAbs were verified using size-exclusion chromatography multi-angle light scattering (SEC/MALS) (**Supplementary Figure S4**).

### Non-reduced and reduced SDS-PAGE of CR3022 and its variants

The size and purity of the CR3022 and its variants were evaluated by sodium dodecyl sulfate-polyacrylamide gel electrophoresis (SDS-PAGE) under non-reduced and reduced conditions. In brief, mAb solution was mixed with LDS sample buffer (Thermo Fisher Scientific Inc., Waltham, MA, USA), and then was incubated at 70°C for 10 min. For the preparation of reduced condition samples, TCEP solution (Thermo Fischer Scientific) was added to half of the samples and then was incubated at room temperature for 5 min. The samples were separated by SDS-PAGE (1 µg/lane) using 4-12% gradient Bis-Tris gels (Thermo Fisher Scientific). The proteins in the gel were detected by Coomassie brilliant blue staining and photographed using a gel imaging system (Chemi-Doc MP, Bio-Rad Laboratories Inc, Hercules, CA, USA).

### ELISA for CR3022 and its variants affinity assessment to RBD and FcRn protein

Recombinant SARS-CoV2 spike RBD glycoprotein (Abcam plc, Cambridge, UK) or recombinant human FcRn protein (R&D Systems, Minneapolis, MN) were coated on the high-binding half-area 96-well plate at 2.5 µg/mL in bicarbonate buffer (pH 9.6) overnight at 4°C. 5% non-fat milk was used for blocking for 1 h at room temperature. CR3022 and its variants diluted in 1% non-fat milk were added to the plate, and then the plates were incubated overnight at 4°C. For the affinity assessment to RBD, PBS at pH 7.4 was utilized to prepare 1% milk, while for the affinity assessment to FcRn, the pH was adjusted to 6.0 by adding hydrochloric acid to PBS. After washing with PBS, goat anti-human IgG Fc horseradish peroxidase (Abcam, 1:5000 dilution) was added to the plate, and then the plates were incubated for 1 h at room temperature. 1-step TMB substrate solution (Thermo Fisher Scientific) was added for the enzymatic degradation of hydrogen peroxide. The reaction was stopped with 1 M hydrochloric acid, and the absorbance at 450 nm was measured using an AccuSkan FC plate reader (Fisher Scientific).

### WD-HAE cell culture

Human primary tracheobronchial airway epithelial cells were obtained from individual donors without documented lung disease including, cystic fibrosis, chronic obstructive pulmonary disease, or asthma. Airway epithelial cells were isolated from surgical specimens by the University of North Carolina Cystic Fibrosis Center Tissue Culture Core Facility using Institutional Review Board-approved protocols. The isolated cells were seeded on type IV collagen-coated polyester filter inserts (pore size, 0,4 µm: area, 1.12 cm^2^; Transwell, Corning, NY, USA) (30) and grown in Pneumacult ALI Basal Medium (StemCell Technologies). After reaching confluence, culture conditions were changed to an air-liquid interface (ALI) and cultured in Pneumacult media containing the recommended supplements to enhance the differentiation of epithelial cells resembling the airway epithelium *in vivo*. After 4 weeks of culture under these conditions, abundant ciliated cells were observed using light microscopy. Transepithelial electrical resistance (TEER) was measured as ≥300 Ω·cm^2^ using an EVOM2 instrument (World Precision Instruments, Sarasota, FL, USA).

### mAb transport experiments using WD-HAE culture

Transport experiments were performed while maintaining ALI conditions. After the apical surface of HAE cells was washed using PBS, CR3022, CR3022-YTE, or CR3022-LS (20 or 200 µg/mL) were applied to the apical side (10 µL diluted in PBS) or basal side (2 mL diluted in medium). For recovery of cell culture apical surface washes, 200 µL of PBS was added for 15 mins and then harvested at designed time points (6, 24, 48, 72, 96 h). For basolateral sampling, 200 µL of media were collected from the basolateral side of WD-HAE at designed time points (6, 24, 48, 72, 96 h) and replaced with equal volumes of media. mAb concentrations in samples were quantified by ELISA using RBD-coated plates as described above. In the inhibition experiments, CR3022 (200 µg/mL) with intravenous immunoglobulin (IVIG, 2 mg/mL) was applied to the basal side (2 mL diluted in medium).

### Bulk RNA sequencing of cultures of large airway epithelium or small airway epithelium

*FCGRT* expression levels were determined in LAE and SAE airway cultures by interrogating a previously published BulkRNASeq database (31). As detailed methods are contained in the original publication, only brief details are supplied here. Matched LAE and SAE cells were isolated from freshly excised normal human lungs obtained from the UNC Tissue and Cell Culture Procurement Core from transplant donors with lungs unsuitable for transplant. Briefly, LAE cells were isolated from the cartilaginous airways by protease digestion and SAE cell isolated from distal lung from the same lung as utilized for LAE cell isolation. Small airways were identified based on the absence of wall cartilage and outer diameters less than 2 mm. After expansion, LAE and SAE were cultured on human placental type IV collagen-coated, 0.4 μm pore size tissue culture membranes for 4 weeks, at which point RNA was extracted from the cells using TRI Reagent (Sigma) according to the manufacturer’s instructions. Total RNA was used for bulk RNA-seq. For Bulk RNAseq library preparation and sequencing, mRNAs were enriched using oligo (dT) beads. The library was sequenced by Novaseq 6000 platform. Raw sequence reads in FASTQ format were mapped to the reference genome GRCh38, and Gencode v32 gene and transcript annotation for exon and splice site mapping, using HISAT2 aligner. Gene expression was estimated by StringTie as Transcript Per Million (mapped reads), or TPM. Gene expression (TPM) was normalized using the voom function from Bioconductor limma package based on mean-variance modeling, and differential expression analysis was performed as linear mixed-effect models using the dream function from the Bioconductor variance Partition package. The linear mixed-effect model applied treated donor code as random effect factor on gene expression. All transcriptome data were deposited in GEO. LAE/SAE bulkRNAseq data were assigned to GSE160675.

### Histological assessment of FcRn expression in WD-HAE cultures and human tracheal airway epithelium

Histological cross-sections of WD-HAE cultures and human tracheal tissues fixed in 10% buffered formalin were generated by the UNC Pathology Services Core. Histological sections were deparaffinized with xylene and ethanol gradients. Primary antibodies were added to tissue sections after heat-mediated antigen retrieval in TRIS-EDTA buffer at pH 9.0 and blocking of non-specific Ig-binding sites with 10% Normal Goat Serum (Jackson labs). A primary rabbit antibody directed against FcRn (Proteintech 16190-1-AP) or as control, an isotype rabbit IgG (Jackson labs) was applied to tissue sections at 1 ug/ml for both antibodies. Following primary antibody incubation overnight, tissue sections were then incubated with fluorescent secondary antibody (anti-rabbit IgG AlexaFluor 594) at a 1:700 dilution (Invitrogen), and stained with DAPI. Tissue sections were imaged on an Olympus VS200 virtual slide scanning system with an Allied Vision Pike 5 CCD progressive scan camera using OlyVia software (version 4.0) and images processed with Adobe Photoshop (CS6).

## Results

### Production & characterization of CR3022 and its variants

**Figure 1:**
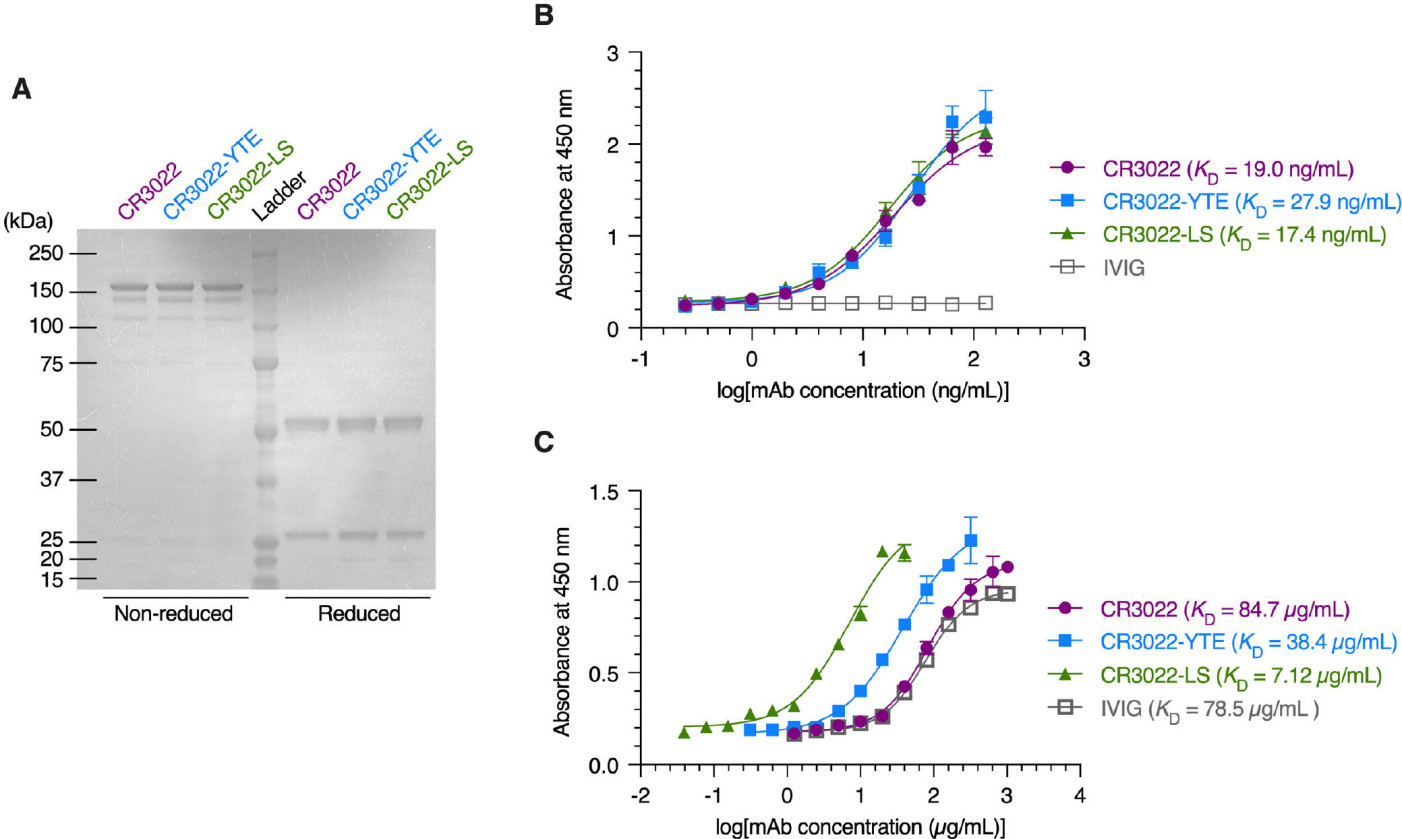
Characterization of the CR3022 and its variants. **(A)** Coomassie brilliant blue staining of non‐reduced and reduced protein gels depicting monoclonal antibodies (mAbs). **(B, C)** ELISA analysis illustrating the binding affinity of mAbs and intravenous immunoglobulin (IVIG) to the SARS‐CoV‐2 spike RBD protein **(B)** and FcRn protein **(C)**. Each data point represents the mean ± standard deviation (n = 3).

The human recombinant monoclonal antibody CR3022 directed against the receptor-binding-domain (RBD) of SARS-CoV-2 Spike and its variants with modified Fc regions (CR3022-YTE and CR3022-LS) were produced by transient transfection of *Nicotiana* plants engineered to express proteins and mAbs decorated with human N-glycosylation (29, 32, 33); the same production system has been used previously to generate mAbs for clinical trials (34, 35). The molecular size and purity of CR3022 and its variants was assessed by sodium dodecyl-sulfate polyacrylamide gel electrophoresis (SDS-PAGE) and all recombinant mAbs showed the expected protein molecular size in non-reduced (150kD) and reduced conditions (50/25kD) (**Figure 1A**). The binding affinities of the mAbs to SARS-CoV2 Spike RBD glycoprotein were measured by antigen-specific enzyme-linked immunosorbent assay (ELISA). CR3022 and its variants all exhibited highly comparable binding affinities to RBD glycoprotein (**Figure 1B**), with measured EC50 representing the equilibrium dissociation constant (*K*_D_) values of ~19, 28, and 17 ng/mL for CR3022, CR3022-YTE, and CR3022-LS, respectively. We also measured the FcRn affinities via ELISA. In good agreement with previously published studies, the YTE and LS mutations of Fc substantially improved the affinity of CR3022 for FcRn compared to the IgG1-Fc: with *K*_D_ values of CR3022, CR3022-YTE, CR3022-LS, and intravenous immunoglobulin (IVIG; included as experimental control) determined as 84.7, 38.4, 7.12, and 78.5 µg/mL, respectively (**Figure 1C**). Altogether, our panel of recombinant CR3022 and its variants all exhibited similar binding affinities to the SARS-CoV-2 Spike RBD, while exhibiting the expected variable affinities to FcRn.

### Basal-to-apical transport of CR3022 and its variants across WD-HAE cultures

We next used WD-HAE cultures to assess the basal-to-apical transepithelial transport of CR3022 and its variants over time. To achieve the typical range of systemic concentrations of antiviral mAbs achieved shortly after dosing [~20-200 μg/mL (36–40)], we introduced either 40 or 400 μg of our mAbs to the 2 mls of basal media. We then assessed the apical levels of CR3022 every 24 hrs by adding 100 microliters of PBS to the apical surfaces of HAE cultures for 15 mins, after which the PBS apical wash was harvested and used to measure the amount of CR3022 using the same highly sensitive RBD-specific ELISA to quantify CR3022. This assay eliminated background signal associated with other antibodies potentially present in serum-supplemented commercial cell culture media.

**Figure 2:**
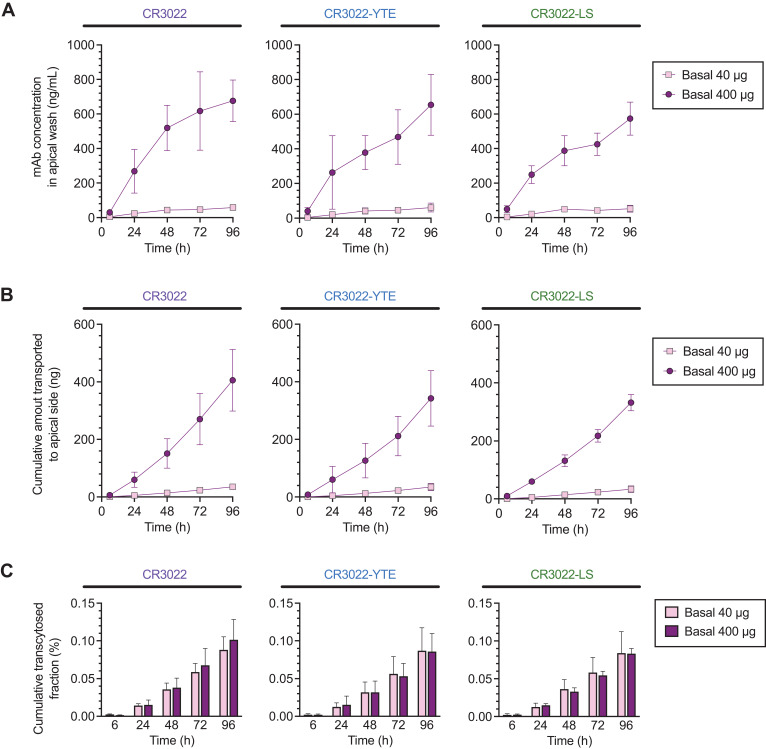
Basal‐to‐apical transport of CR3022 and its variants across WD‐HAE cell culture. **(A)** The mAb concentration in the apical wash over time at basal doses of 40 and 400 µg. **(B)** The cumulative amount of mAbs transported to the apical side over time at basal doses of 40 and 400 µg. **(C)** Cumulative transcytosed fraction based on the applied dose to the basal side over time. Each point represents the mean ± standard deviation (n = 3 for each condition/ID, total of three donor IDs).

The concentrations of CR3022 and its variants in the apical washes increased over time in a dose-dependent manner. While the low mAb dose resulted in detection of low concentrations of each mAb (less than 40 ng/mL) in the apical wash at each time point, the high mAb dose resulted in appreciable quantities of mAbs in the apical washes been detected reaching hundreds of ng/mL (**Figure 2A**; **Supplementary Figure S1A**). Surprisingly, we found comparable levels of CR3022, CR3022-YTE and CR3022-LS in the apical washes over time, suggesting that all our mAbs constructs exhibited similar transepithelial transport rates regardless of the differences in affinity for FcRn. The cumulative transcytosed amount over the first 4 days were consistent between all 3 mAbs, reaching ~40 ng and ~400 ng total over the first 4 days post-dosing with the low and high mAb dose, respectively (**Figure 2B**; **Supplementary Figure S1B**). However, relative to the actual concentration of mAbs in the basal media, the total fraction of mAb transcytosed over the first 4 days remained exceptionally low, with roughly 0.1% of the total mAb dose transcytosed for each of the three mAbs (**Figure 2C**). Overall, the mAb concentration in apical washes over time, the cumulative amount of mAb transcytosed, and the cumulative fraction of mAbs transcytosed were not statistically different between CR3022, CR3022-YTE and CR3022-LS (see **Supplementary Table 1** for statistics summary). The cumulative fraction of the mAbs transcytosed were also similar between the two different mAb doses. These results highlight the limited capacity of mAb in the basal compartment to cross the airway epithelium into the apical compartment, and that the basal-to-apical transport rate of mAbs are not dependent the affinity of mAb to FcRn.

### Involvement of FcRn in basal-to-apical transport of mAbs

**Figure 3:**
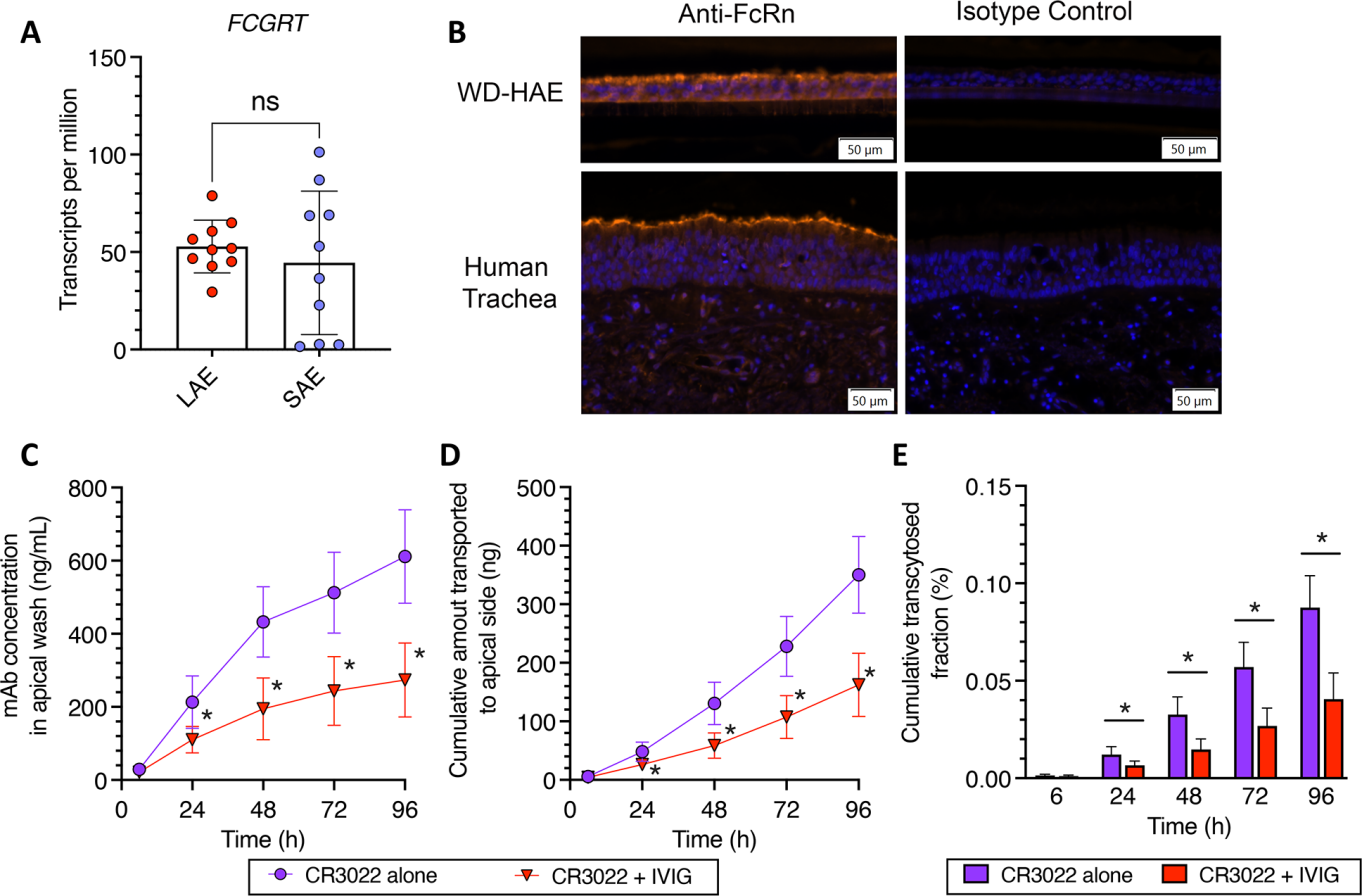
**(A)** BulkSeq of cultures of Large Airway Epithelium (LAE) and Small Airway Epithelium (SAE). **(B)** Immunohistochemical detection of FcRn proteins in WD‐HAE cultures and human trachea, compared to isotype staining control. **(C–E)** Effect of IVIG on the basal‐to‐apical transport of CR3022 through WD‐HAE cell culture. **(C)** The mAb concentration in the apical wash over time at basal doses of 400 μg, with or without co‐application of IVIG (4 mg). **(D)** The cumulative amount of mAb transported to the apical side over time at basal doses of 400 μg with or without IVIG (4 mg). **(E)** Cumulative transcytosed fraction, calculated based on the applied dose to the basal side, over time with or without IVIG. Each point represents the mean ± standard deviation (n = 3 for each condition/ID, a total of three donor IDs). *p < 0.01: indicates statistical significance compared to CR3022 alone, using Bonferroni‐corrected t‐test. “ns” stands for “Not significant”.

As we did not detect enhanced transcytosis of both CR3022-YTE and CR3022-LS, we sought to confirm FcRn was present in the WD-HAE cultures. We did so by first assessing the expression of *Fc Gamma Receptor and Transporter* (*FCGRT*), the gene encoding FcRn, in WD-HAE cultures. We note that WD-HAE cultures in our studies utilized cells harvested from the large cartilaginous proximal airways. Thus, we first interrogated published Bulk RNA sequencing data (BulkRNAseq) previously generated at UNC on WD-HAE cultures derived from large airway epithelial (LAE) cells in 10 individual tissue donors (31), and found *FCGRT* to be abundantly expressed in WD-HAE from all 10 tissue donors (**Figure 3A**).

The previously published BulkRNASeq study also had data from small non-cartilaginous airway epithelium (SAE) from the same 10 tissue donors, which provided a unique opportunity to compare *FCGRT* expression levels in LAE and SAE derived from the same tissue donor. While *FCGRT* expression was similar in magnitude in SAE as in LAE, the expression levels in SAE were more variable, with 7 of 10 tissue donors expressing *FCGRT* in SAE at levels comparable to that in LAE (**Figure 3B**).

To further ascertain expression of the FcRn protein in WD-HAE, we performed immunohistochemistry on histologic sections of WD-HAE and human tracheal tissue (**Figure 3B**). Using immunofluorescence as our detection method, we found the airway epithelial cells in WD-HAE cultures were strongly positive for FcRn immunoreactivity, with more robust signal at the apical aspects of the luminal epithelial cells. Similarly, in sections of human tracheal tissue, FcRn immunoreactivity was largely found in the epithelial cells with increased immunoreactivity at the apical aspects of the epithelium. These results clearly indicate that FcRn is highly expressed in WD-HAE cultures, and thus the failure to observe increased transcytosis with Fc variants is not due to the absence of FcRn in our WD-HAE culture model.

To functionally assess the involvement of FcRn in basal-to-apical transcytosis in WD-HAE cultures, we next performed a competitive inhibition experiment using 10-fold excess of a clinical preparation of human IgG (4 mg of intravenous immunoglobulin (IVIG)) to the high dose of CR3022 (400 μg). While we were able to detect statistically significant differences in CR3022 concentrations in apical washes by 24 hrs after dosing (**Figures 3C–E**), the reduction in the amount of CR3022 transcytosed was markedly less than expected from the stoichiometric ratio of input IVIG vs. CR3022. Specifically, CR3022 concentrations and cumulative amount transcytosed were only decreased by ~50% in the presence of 10-fold excess IVIG, which should have reduced apical CR3022 by ~90% if FcRn-mediated transcytosis is the sole mechanism of basal-to-apical transport. These results suggest FcRn likely has a significant but not exclusive role in the basal-to-apical transcytosis of mAbs across WD-HAE cultures.

To ascertain the impact of having excess IVIG in the environment, we performed additional studies with the YTE and LS mAb variants in the presence of excess IVIG competition (**Supplementary Figure S2**). Unlike with wildtype CR3022, excess IVIG reduced the two mAbs to even less extent, with virtually no detectible difference in the amount of CR3022-YTE that underwent basal-to-apical transcytosis, and only a very modest (~20%) reduction in the amount of CR3022-LS that underwent basal-to-apical transcytosis. These results are consistent with a model where greater FcRn affinity does not increase kinetics or rate of FcRn transcytosis. It suggests that, in the presence of high IVIG, increased FcRn affinity may allow mAbs to better compete for available FcRn and very modestly increase basal-to-apical transport.

### Apical-to-basal transport of CR3022 and its variants across WD-HAE cultures

Direct inhaled delivery of mAbs is quickly becoming a reality for treatment of acute respiratory infections, as illustrated by our recent Phase 1 study of an inhaled antiviral mAb therapy for COVID-19 (41) and recent large animal studies for RSV and SARS-CoV-2 (42, 43). This motivated us to determine how quickly our mAbs delivered to the apical surface of the airway would undergo apical-to-basal transport across HAE. To test this, we added either 0.2 or 2 μg of CR3022 or each of the CR3022 variants directly to the apical compartment of WD-HAE, then quantified the concentrations of mAbs in the basal media over time by sampling a small fraction of the total volume of media.

**Figure 4:**
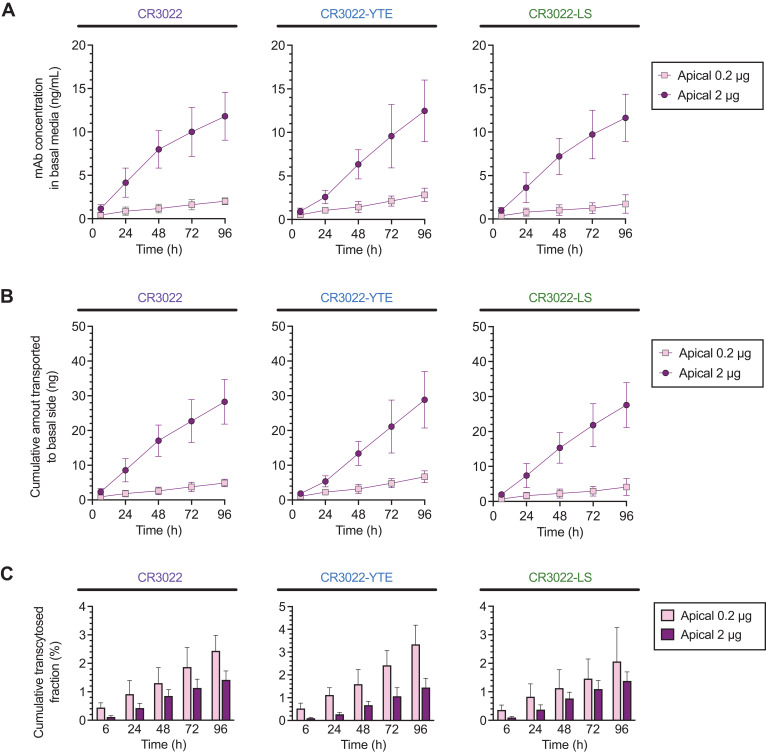
Apical‐to‐basal transport of CR3022 and its variants across WD‐HAE cell culture. **(A)** The mAb concentration in the basal media over time at apical doses of 0.2 and 2 µg. **(B)** The cumulative amount of mAbs transported to the basal side over time at apical doses of 0.2 and 2 µg. **(C)** Cumulative transcytosed fraction based on the applied dose to the apical side over time. Each point represents the mean ± standard deviation (n = 3 for each condition/ID, a total of three donor IDs).

Similar to the earlier basal-to-apical transport study above, we found limited distribution of mAbs into the basal media over the first 4 days, with concentrations reaching only ~1-2 ng/mL 96 hrs following apical dosing of 0.2 μg of CR3022 (**Figure 4A**; **Supplementary Figure S3A**). At the higher apical mAb dose of 20 μg CR3022, we saw concentrations in basal media reaching ~10-15 ng/mL (**Figure 4A**), roughly proportional to the 10-fold greater input mAb dose. At both input doses, CR3022 and its YTE and LS variants exhibited highly comparable cumulative fraction of mAb transcytosed to the basal media (**Figure 4B**; **Supplementary Figure S3B**). When we adjust for the input dose, the cumulative fraction of mAb transcytosed over the first 4 days reached ~1-3% for all three mAbs at the high input dose (**Figure 4C**); for CR3022 and CR3022-YTE but not CR3022-LS, the cumulative fraction transcytosed at the lower dose was even higher, although the difference did not reach statistical significance (see summary statistics in **Supplementary Table S2**). Indeed, similar to the studies on basal-to-apical mAb distribution, there was overall limited distribution of mAbs from the apical surface to the basal compartment, and no statistically significant differences were found between the three mAbs tested. The results here are consistent with the limited distribution of mAbs into the submucosa and the systemic circulation following nebulized inhaled delivery in our Phase 1 study. These results here also suggest minimal dependence of FcRn-affinity on apical-to-basal transport of mAbs across human airway epithelium.

### Comparing efficiencies of apical vs. basal dosing in HAE

**Figure 5:**
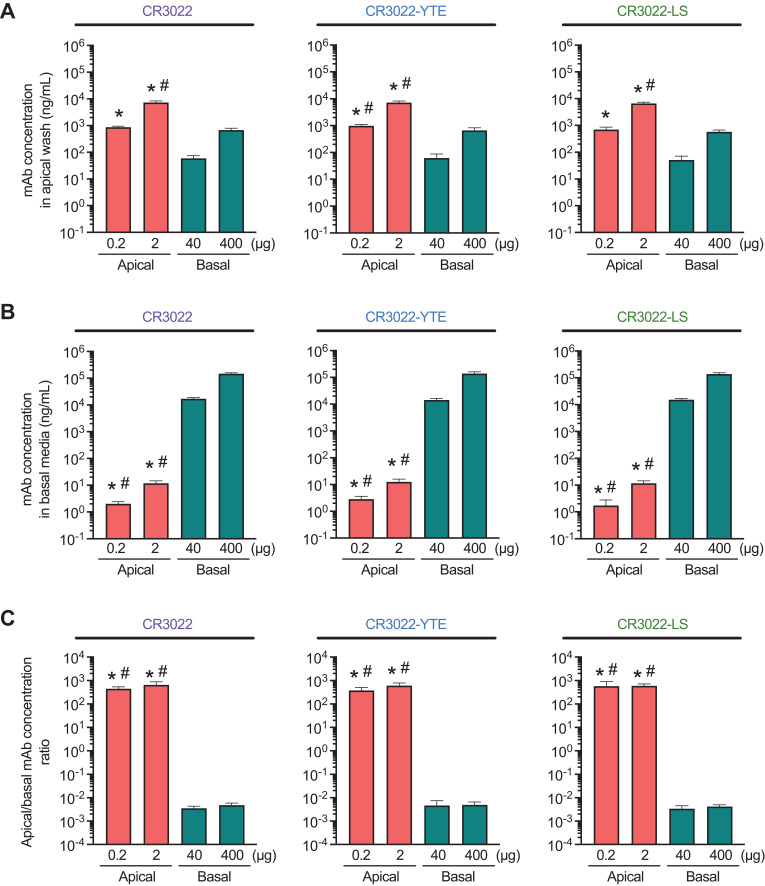
Comparative analysis of apical **(A)** and basal **(B)** concentrations of CR3022 and its variants after 96 h of apical (0.2 or 2 µg) or basal (40 or 400 µg) application on WD‐HAE cell culture. **(C)** The ratio of mAb concentrations in the apical/basal compartment. Each point shows an average between three donor IDs. Each point represents the mean ± standard deviation (n = 3 for each condition/ID, a total of three donor IDs). *p < 0.01 indicates statistical significance compared to the basal dose of 40 µg. #p < 0.01 indicates statistical significance compared to the basal dose of 400 µg. Statistical comparison was based on one‐way ANOVA.

Finally, we sought to directly compare the concentrations in the apical and basal compartments of the three CR3022 mAb variants following apical vs. basal delivery. Not surprisingly, the mAb concentrations in the apical compartment remained high following apical delivery, with mAb concentrations reaching ~1 and ~10 μg/mL in the apical wash for the 0.2 and 2 μg doses, respectively. For mAbs dosed into the basal compartment to reach the same concentration in apical washes as the low apical mAb dose, a ~2000-fold increase in CR3022 (i.e. 400 μg) was necessary (**Figure 5A**). The superiority of direct pulmonary delivery is also illustrated by the fact that a 200-fold lower mAb dose delivered apically still achieved ~10-fold greater mAb concentrations in the apical washes (p<0.01); the much greater mAb levels following apical dosing were consistent regardless of the FcRn-affinity of the various CR3022 mAbs.

We next compared the concentration of CR3022 mAbs in the basal compartment. In good agreement with our expectations, the basal concentration of CR3022 administered apically was over three orders of magnitude lower than when the same mAbs were applied to the basal side, albeit at a much higher dose (**Figure 5B**). Given the vast difference in the amount of mAbs dosed apically *vs.* basally, we estimated the delivery efficiency based on the ratios for the apical *vs.* basal mAb concentrations (**Figure 5C**). Apical delivery of mAb, regardless of the mAb FcRn-affinity and the actual dose, achieved an apical:basal ratio of ~500:1 (i.e. the concentration in apical washes is ~500-fold higher than in basal media). In contrast, basal delivery resulted in a ratio close to 1:500 regardless of the FcRn-affinity and the actual dose of the mAbs. The difference in dosing route yields a nearly 5-log difference in distribution efficiency. Since viruses that cause acute respiratory infections are predominantly present in the airway lumen, achieving effective inhibitory concentrations of mAb on the airway luminal surface is essential for maximizing therapeutic efficacy. Our results underscore the route of mAb dosing, rather than Fc engineering, as the key to achieving high apical concentrations as part of the intervention against respiratory viruses early in infection.

## Discussion

While the COVID-19 pandemic has advanced the development of numerous vaccines and antivirals, a significant number of viral respiratory pathogens do not have licensed vaccines or antivirals available, including human metapneumoviruses, parainfluenza viruses, adenoviruses, rhinoviruses, and seasonal coronaviruses. Given the exceptional potencies and safety of mAb as a class of antivirals, highlighted by multiple mAbs receiving EUA as the first antivirals to prevent severe COVID-19 in high risk individuals with early to moderate COVID-19, we believe mAbs overall represent an ideal class of antivirals for early intervention against commonly circulating respiratory viruses (10). Nonetheless, key attributes in mAb engineering and delivery that could maximize their efficacy against viral respiratory infections are not well defined. Here, by utilizing mAbs with engineered Fc regions and WD-HAE cultures that recapitulate the morphology and functionality of the human respiratory epithelium, we showed that enhanced FcRn-affinity of mAbs does not markedly increase the rates of basal-to-apical mAb transcytosis, which is likely limited by the actual abundance and rates of FcRn transcytosis. As a result, only a small fraction of the total dose of mAb (~0.2%) delivered to the basolateral compartment transcytosed to the apical surface during the first 4 days. Given the exceedingly limited transcytosis, the route of dosing (apical *vs.* basal) plays a far more influential role in achieving highly inhibitory levels of mAb in the airways quickly. Our findings have important ramifications on the development of mAbs as interventions against various viral respiratory infections, and strongly motivate the development of inhaled mAb formulations for treatment and development of parenteral formulations for prophylaxis.

Various mAbs against SARS-CoV-2, including REGEN-COV (44), bamlanivimab and etesevimab (45), Evusheld (tixagevimab/cilgavimab) (24), Xevudy (sotrovimab) (26) and Regkirona (regdanvimab) (46), all achieved impressive efficacy ranging from 70-85% effectiveness in preventing hospitalization, despite the limited distribution into the respiratory tract following systemic dosing. In contrast, all mAbs targeted against influenza virus that have advanced into Phase 2 studies to date have failed to achieve meaningful clinical benefit, including CR8020 (47), MEDI-8852 (48), MHAA4549A (49) and most recently VR-2482. Since SARS-CoV-2 and influenza viruses both infect and spread via the apical surfaces of the epithelium, we believe the differences in these outcomes are likely attributed to the excess of mAb concentration relative to the inherent inhibitory activities of the mAbs. SARS-CoV-2 mAbs exhibit IC50s typically in the range of 1-50 ng/mL; implying the apical mAb concentrations observed with the high mAb dose (hundreds of ng/mL) are likely sufficient to confer strong inhibition of SARS-CoV-2 replication. In contrast, as the IC50s of most broadly neutralizing influenza mAbs are in the range of ~1-10 μg/mL, it is likely that despite the very high mAb doses used, the mAb concentrations in the airway lumen never reached levels required to confer strong inhibition of spread of influenza virus infection.

Treatment of acute viral respiratory infections is highly dependent on early intervention and antiviral therapies initiated too late in the course of infection are unable to reverse the infection-induced inflammation that is responsible for significant pulmonary morbidities. This implies achieving highly inhibitory concentrations of antivirals in the early stages of infection is essential for preventing the progression of viral infection from the URT to the LRT. Direct delivery of mAbs to the airway surface via inhalation enables very high mAb concentrations well in excess of the inhibitory threshold to be achieved nearly instantaneously, while using only a fraction of the total mAb dose given by systemic dosing. Indeed, compared to basolateral delivery, a 2,000-fold lower mAb dose delivered apically achieves the same mAb concentration on the apical surface in this study, regardless of the FcRn affinity. These *in vitro* findings are consistent with those from our recent Phase 1 study showing once-per-day dosing of inhaled mAb achieved lumenal concentrations more than 100,000 times the IC50 at Cmax, and still 100+ fold above the IC50 at trough. The far more efficient pulmonary dosing with inhaled mAb delivery is expected to lead to far more attractive pharmacoeconomics, enable treatment of more patients with less drug, and further improve the already excellent safety profile of antiviral mAbs (50).

Typically, IgG_1_ exhibits a serum half-life of ~ 20-25 days; mAbs engineered with greater FcRn affinity can circulate appreciably longer, with half-lives often extending to ~50-70+ days (27). The exceptionally long systemic half-lives of mAbs implies that no more than ~2% of the total dose can be lost from the blood per day. Simple mass-balance analysis imposes that the fraction of systemically circulating mAb with typical IgG1-Fc that are lost to the respiratory tract each day must be even smaller (i.e. a fraction of a percent) relative to the total dose. For mAbs with greater FcRn affinity leading to even lower rates of loss from the circulation, the amount that could be lost to the respiratory tract as a fraction of the total dose must be comparably low, as greater loss due to pulmonary distribution would actually limit the prolonged circulation. This in turn implies that mAbs with greater FcRn affinity is unlikely to achieve greater local C_max_ in the respiratory tract than conventional IgG_1_ mAbs early on, in good agreement with our measurements of mAb concentrations on the apical surface. Altogether, the limited basal-to-apical transcytosis of parentally dosed mAbs into the respiratory tract, as well as the seeming lack of dependence on FcRn affinity, are both consistent with the prolonged systemic circulation of mAbs.

While the increased affinity of Fc for FcRn did not greatly enhance the rate of transcytosis of mAbs across human airway epithelium over short time scales (days), their prolonged systemic circulation means that there is much higher levels of mAb retained in the blood over longer time scales (months). Assuming mAb levels in the lung are at a steady state with mAb levels in the blood, the concentration of mAb in airway secretions should be proportionally greater over long time scales. This suggests that mAbs engineered with increased FcRn affinity should provide more effective prophylaxis over mAbs with typical FcRn affinities (IgG1-Fc) not because it leads to greater rates of mAb transcytosis into the apical secretions, but rather simply due to their prolonged systemic circulation. This aligns well with the observation that, while nirsevimab (containing YTE-mutations) and motavizumab (wildtype IgG1-Fc) both show similar prophylactic efficacies (both mAbs reduced medically-attended LRT infections by ~70%) (51), nirsevimab was able to achieve the protection with just a single dose per RSV season *vs.* monthly motavizumab injections (27).

There are significant advantages to advancing the same mAb molecule for both passive immunization (given systemically) and early intervention to prevent spread of infection (given by inhalation). These include costs and time savings associated with eliminating redundant preclinical activities, as well as greater market reach by addressing two distinct markets with a single product. Our work here shows that CR3022 variants with increased affinity for FcRn do not lead to greater basal-to-apical or apical-to-basal transcytosis. Hence, to maximize potential clinical usage of the same molecule, our work suggests mAbs with greater FcRn affinity should be advanced for both systemic passive immunization and inhaled treatments.

Other studies have investigated apical-to-basal transport of IgG in polarized Caco-2 (52) and T84 (53) models of the intestinal epithelium. Transcytosis was completely inhibited when the Fc affinity for FcRn was abolished, whereas a 20-fold improvement in Fc affinity to FcRn (K_D_ value) increased the transport by only 1.6-fold. While other reports have implicated the involvement of FcRn in IgG transcytosis using respiratory epithelial cells from the URT (54) and the LRT (55), there have been no reported instances of a comparative study on the transepithelial transport of engineered mAbs with altered FcRn affinity. Our results here provide the first insights into whether FcRn affinity influences the rate of transcytosis of mAbs through WD-HAE reflective of the epithelium of the human conducting airways. While the reason for the differential transcytosis results of mAbs with varying FcRn affinities between intestinal and respiratory epithelia remains unclear, it could be influenced by factors such as anatomical tissue differences, variations in FcRn expression, or the turnover of airway mucus secretions produced on the apical surface. In non-human primates, intravenous dosing of MEDI-524 (human IgG1) and its YTE variant saw roughly two-fold more YTE variant in bronchoalveolar lavage fluids (BALF) several days later (56). The findings were frequently interpreted as greater FcRn affinity leading to greater transcytosis, even though the ratios of concentration between BALF and plasma for both mAbs were similar (56). Thus, those results could also reflect similar transcytosis rates with both mAbs, particularly since human IgG1 exhibits a far shorter half-life in monkeys.

The limited pursuit of inhaled delivery of mAbs over the past 2 decades can be attributed in large part to concerns regarding mAb aggregation and loss of binding activity post-nebulization (57, 58). These issues are particularly pronounced with jet nebulizers and ultrasonic nebulizers, where droplet recirculation, thermal stress, and shear stress can promote molecular aggregation, making effective nebulization challenging (59, 60). However, with advances in vibrating mesh nebulizer technology and suitable formulation optimization, it is now possible for mAbs to be stably and efficiently aerosolized without inducing loss of their activity or causing aggregation (43, 61, 62). Nebulized mAb delivery is expected to have an outstanding safety profile (8, 41, 63), while also offering the convenience of at-home dosing, and reduced burden on the healthcare system. Given the advantages in pulmonary distribution, we anticipate inhaled delivery of mAbs would become the preferred approach for treating a variety of acute viral respiratory infections.

## Conclusion

We found here that enhancing the affinity of the Fc region of the mAb did not result in appreciable changes in either the basal-to-apical or apical-to-basal transcytosis of mAb in WD-HAE cultures. Even without greater rates of transcytosis, mAbs with increased FcRn affinity are well suited to enable prolonged prophylaxis. In contrast, given the very slow rates of transcytosis measured, achieving highly inhibitive concentrations of mAb in the airways quickly will likely require direct inhaled dosing. This study provides the blueprint for tuning FcRn affinity in engineering mAb interventions against viral respiratory infections.

## Data Availability

The raw data supporting the conclusions of this article will be made available by the authors, without undue reservation.
