# Supplementary material for: Impact of mAb-FcRn affinity on IgG transcytosis across human well-differentiated airway epithelium
Source: Front Immunol. 2024 Sep 16;15:1371156. doi: 10.3389/fimmu.2024.1371156 (PMC11439726; doi:10.3389/fimmu.2024.1371156)
Supplement: Supplementary file 1 [file DataSheet1.pdf]

## Supplementary Materials

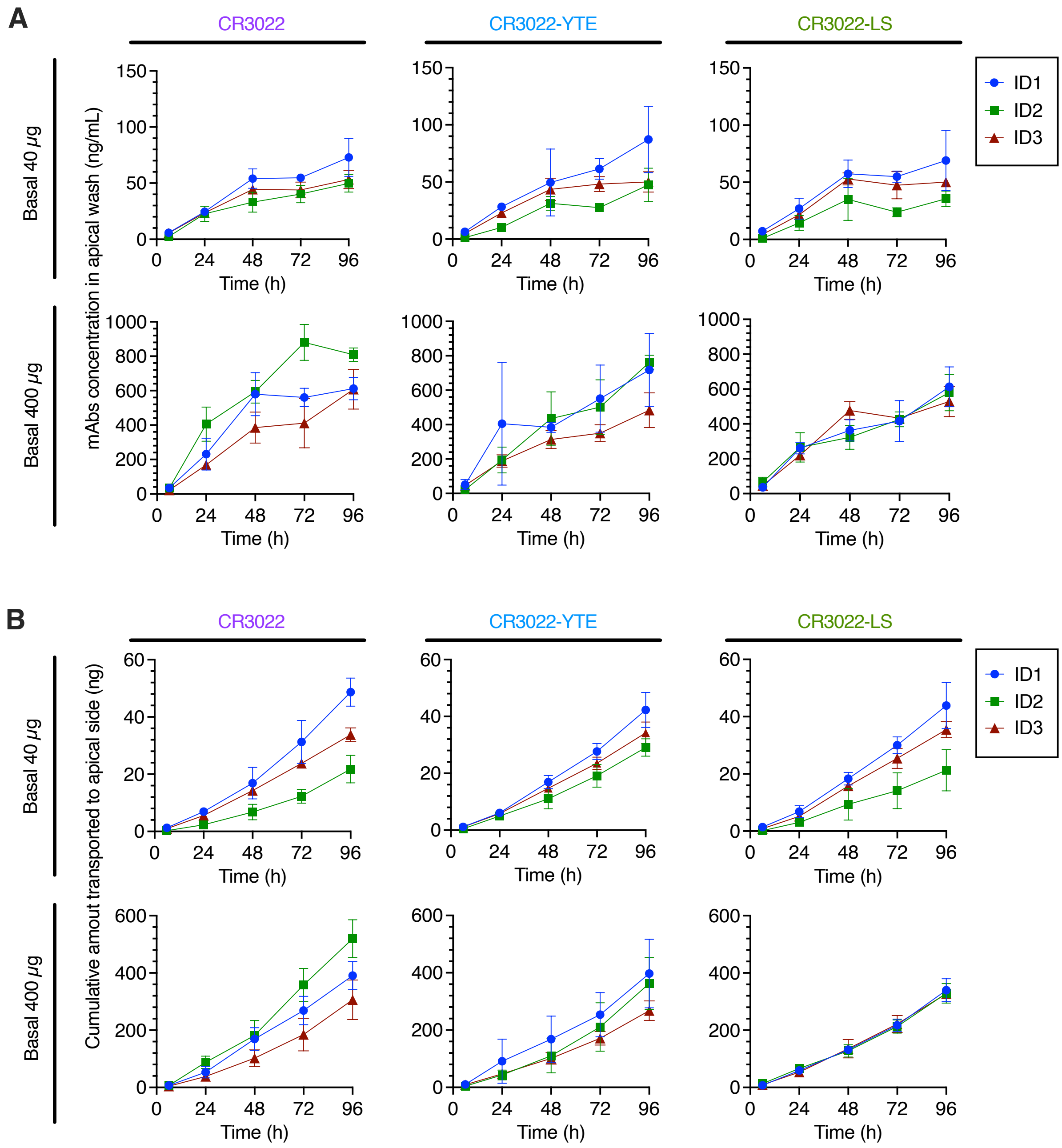


**Figure S1.** Patient ID-based comparison of basal-to-apical transport of CR3022 and its variants across WD-HAE cell culture **(A)** The mAb concentration in the apical wash over time at basal doses of 40 and 400 µg. **(B)** The cumulative amount of mAbs transported to the apical side over time at basal doses of 40 and 400 µg.

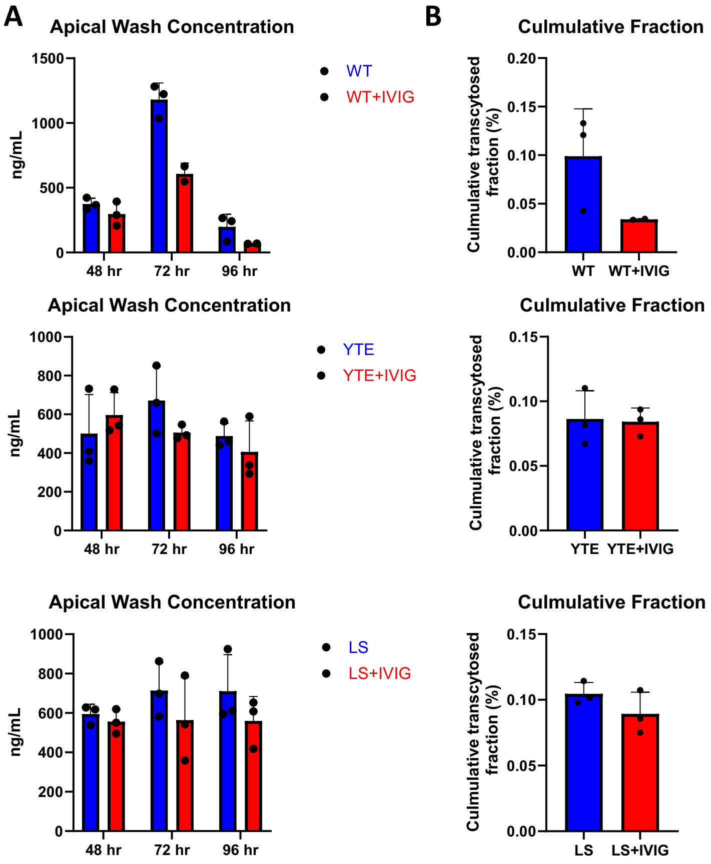


**Figure S2.** Effect of IVIG on the basal-to-apical transport of CR3022, CR3022-YTE and CR3022-LS across WD-HAE cell culture. **(A)** The concentration of various CR3022 variants in the apical wash on Day 2, Day 3 and Day 4 following basal dosing of 400 µg, with or without co-application of IVIG (4 mg). **(B)** The cumulative amount of CR3022 variants transported to the apical side over 4 days, with or without IVIG (4 mg).

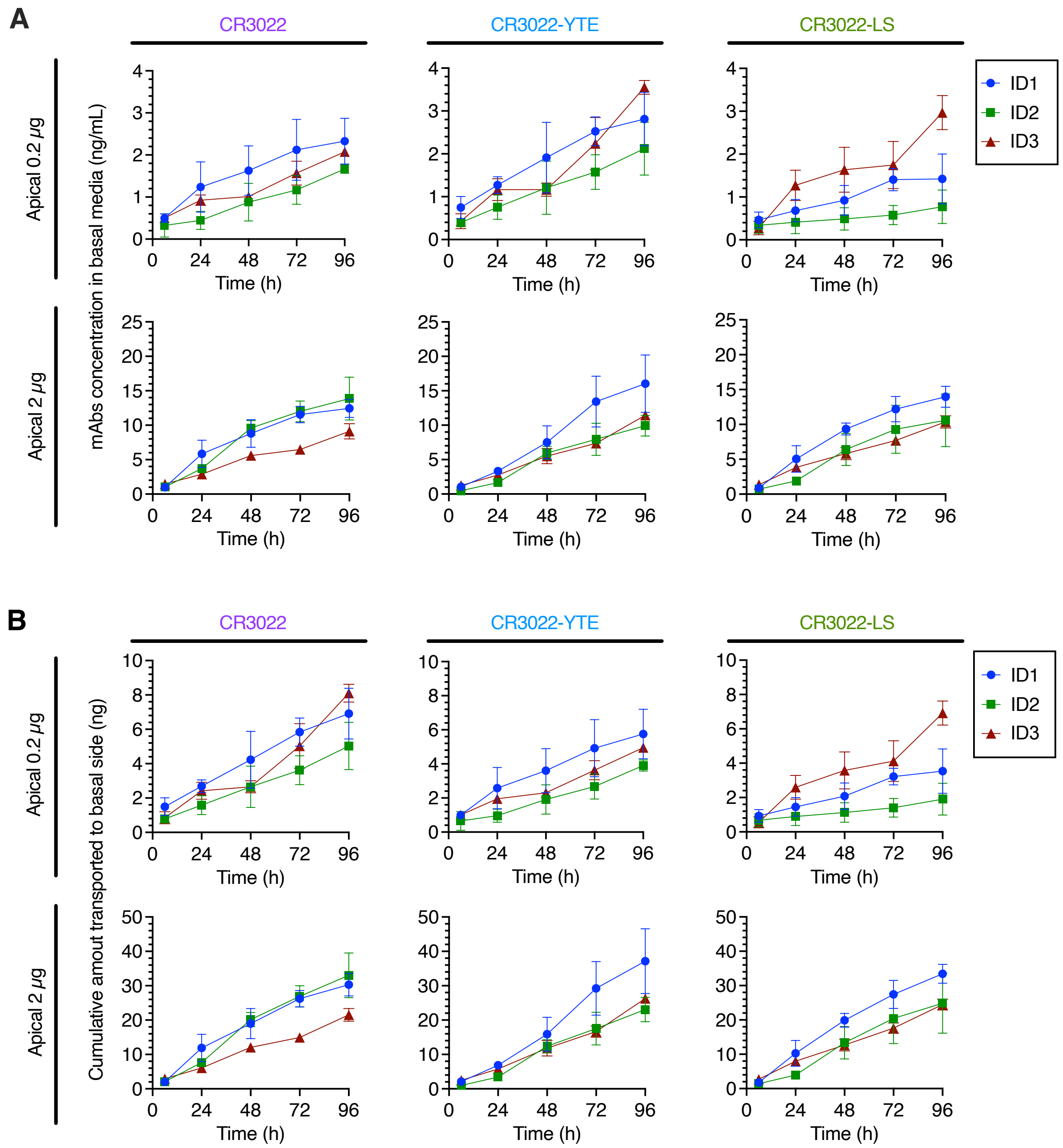


**Figure S3.** Patient ID-based comparison of apical-to-basal transport of CR3022 and its variants across WD-HAE cell culture **(A)** The mAb concentration in the basal media over time at apical doses of 0.2 and 2 µg. **(B)** The cumulative amount of mAbs transported to the basal side over time at apical doses of 0.2 and 2 µg.

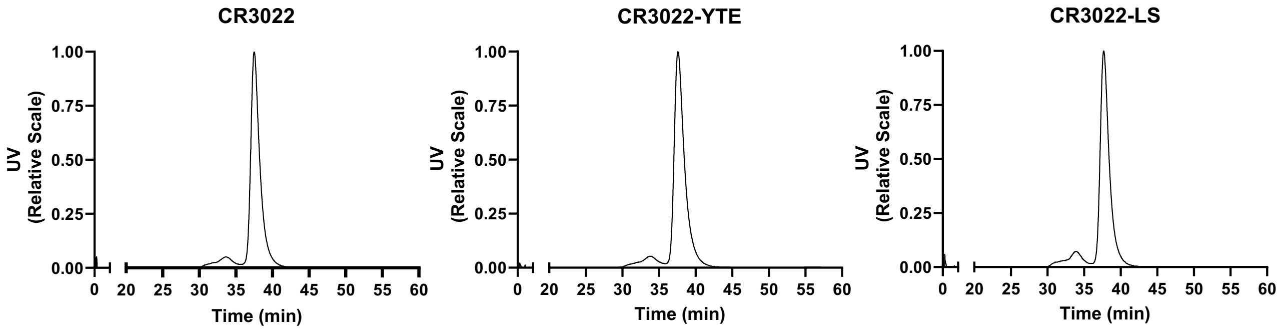


**Figure S4:** Size-exclusion chromatography multi-angle light scattering (SEC/MALS) assessment of purity of CR3022 variants. The % of the UV corresponding to the monomer peak is 91.3, 88.4, and 91.0% for CR3022, CR3022-LS, and CR3022-YTE respectively.

| mAb | Dose (*μ*g) | Time (h) | *P* values for mAb concentration in apical wash | | *P* values for cumulative amount transported | |
|---|---|---|---|---|---|---|
| | | | *vs* CR3022-YTE | *vs* CR3022-LS | *vs* CR3022-YTE | *vs* CR3022-LS |
| CR3022 | 20 | 6 | 0.949 | 0.944 | 0.951 | 0.944 |
| | | 24 | 0.635 | 0.719 | 0.704 | 0.765 |
| | | 48 | 0.938 | 0.794 | 0.759 | 0.992 |
| | | 72 | 0.994 | 0.790 | 0.957 | 0.998 |
| | | 96 | 0.955 | 0.748 | 0.996 | 0.940 |
| CR3022-YTE | 20 | 6 | | 1.000 | | 1.000 |
| | | 24 | | 0.990 | | 0.994 |
| | | 48 | | 0.589 | | 0.688 |
| | | 72 | | 0.844 | | 0.974 |
| | | 96 | | 0.572 | | 0.967 |
| CR3022 | 200 | 6 | 0.484 | 0.078 | 0.484 | 0.078 |
| | | 24 | 0.997 | 0.957 | 0.998 | 1.000 |
| | | 48 | 0.026* | 0.039* | 0.518 | 0.660 |
| | | 72 | 0.151 | 0.051 | 0.165 | 0.224 |
| | | 96 | 0.936 | 0.260 | 0.275 | 0.179 |
| CR3022-YTE | 200 | 6 | | 0.514 | | 0.513 |
| | | 24 | | 0.977 | | 0.998 |
| | | 48 | | 0.981 | | 0.971 |
| | | 72 | | 0.845 | | 0.982 |
| | | 96 | | 0.427 | | 0.963 |

**Table S1.** Summary statistics of basal-to-apical transport experiments of CR3022 and its variants across WD-HAE cell culture. One-way ANOVA analysis was performed using the data in Figure 2. *$p < 0.05$: indicates a statistical significance difference.

| mAb | Dose ($\mu$g) | Time (h) | *P* values for mAb concentration in basal media | | *P* values for cumulative amount transported | |
|---|---|---|---|---|---|---|
| | | | *vs* CR3022-YTE | *vs* CR3022-LS | *vs* CR3022-YTE | *vs* CR3022-LS |
| CR3022 | 20 | 6 | 0.912 | 0.507 | 0.656 | 0.659 |
| | | 24 | 0.802 | 0.999 | 0.581 | 0.895 |
| | | 48 | 0.813 | 0.993 | 0.596 | 0.823 |
| | | 72 | 0.580 | 0.928 | 0.224 | 0.417 |
| | | 96 | 0.503 | 0.999 | 0.105 | 0.659 |
| CR3022-YTE | 20 | 6 | | 0.292 | | 0.204 |
| | | 24 | | 0.825 | | 0.330 |
| | | 48 | | 0.871 | | 0.274 |
| | | 72 | | 0.369 | | 0.017* |
| | | 96 | | 0.531 | | 0.016* |
| CR3022 | 200 | 6 | 0.416 | 0.610 | 0.416 | 0.610 |
| | | 24 | 0.078 | 0.709 | 0.073 | 0.694 |
| | | 48 | 0.200 | 0.675 | 0.163 | 0.650 |
| | | 72 | 0.953 | 0.980 | 0.874 | 0.959 |
| | | 96 | 0.889 | 0.992 | 0.986 | 0.975 |
| CR3022-YTE | 200 | 6 | | 0.942 | | 0.942 |
| | | 24 | | 0.316 | | 0.313 |
| | | 48 | | 0.632 | | 0.586 |
| | | 72 | | 0.994 | | 0.974 |
| | | 96 | | 0.830 | | 0.926 |

**Table S2.** Summary statistics of apical-to-basal transport experiments of CR3022 and its variants across WD-HAE cell culture. One-way ANOVA analysis was performed using the data in Figure 4. *$p < 0.05$: indicates a statistical significance difference.
